# Supplementary material for: Heteropolyacids@Silica Heterogeneous Catalysts to Produce Solketal from Glycerol Acetalization
Source: Nanomaterials (Basel). 2024 Apr 23;14(9):733. doi: 10.3390/nano14090733 (PMC11085283; doi:10.3390/nano14090733)
Supplement: Supplementary file 1 [file nanomaterials-14-00733-s001.zip › nanomaterials-2942492-supplementary.pdf]

# Heteropolyacids@Silica Heterogeneous Catalysts to Produce Solketal from Glycerol Acetalization

**Catarina N. Dias [1], Isabel C. M. S. Santos-Vieira [2], Carlos R. Gomes [3], Fátima Mirante [1,*] and Salete S. Balula [1,*]**

[1] LAQV/REQUIMTE—Laboratório Associado para a Química Verde e Departamento de Química e Bioquímica & Faculdade de Ciências, Universidade do Porto, 4169-007 Porto, Portugal

[2] CICECO—Aveiro Institute of Materials, Department of Chemistry, University of Aveiro, 3810-193 Aveiro, Portugal

[3] CIIMAR—Centro Interdisciplinar de Investigação Marinha e Ambiental & Faculdade de Ciências, Universidade do Porto, 4169-007 Porto, Portugal

* Correspondence: fatima.mirante@fc.up.pt (F.M.); sbalula@fc.up.pt (S.S.B.)

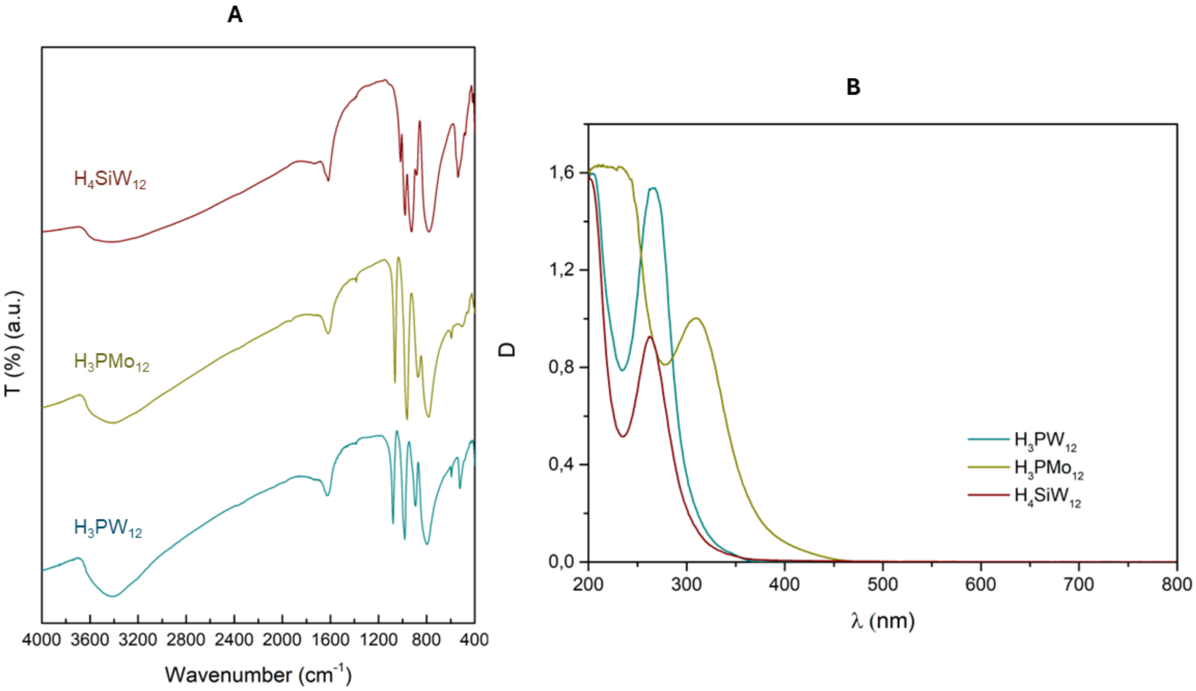


**Figure S1. (A)** FTIR spectra of $H_3PMo_{12}$, $H_3PW_{12}$ and $H_4SiW_{12}$. **(B)** UV-Vis absorption spectra of $H_3PMo_{12}$, $H_3PW_{12}$ and $H_4SiW_{12}$, in acetonitrile.

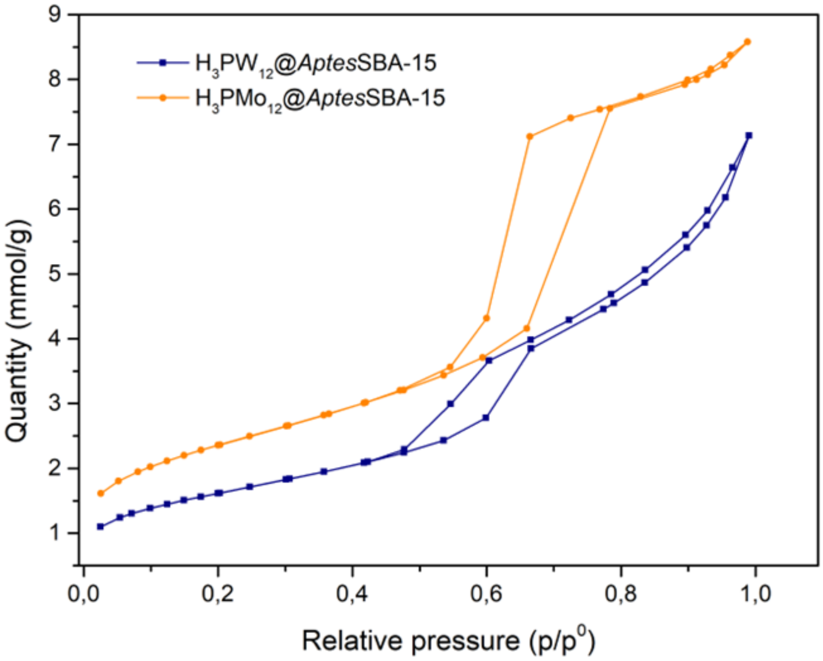


**Figure S2.** Nitrogen adsorption-desorption isotherms of the prepared $H_3PW_{12}$@*Aptes*SBA-15 and $H_3PMo_{12}$@*Aptes*SBA-15 composites.

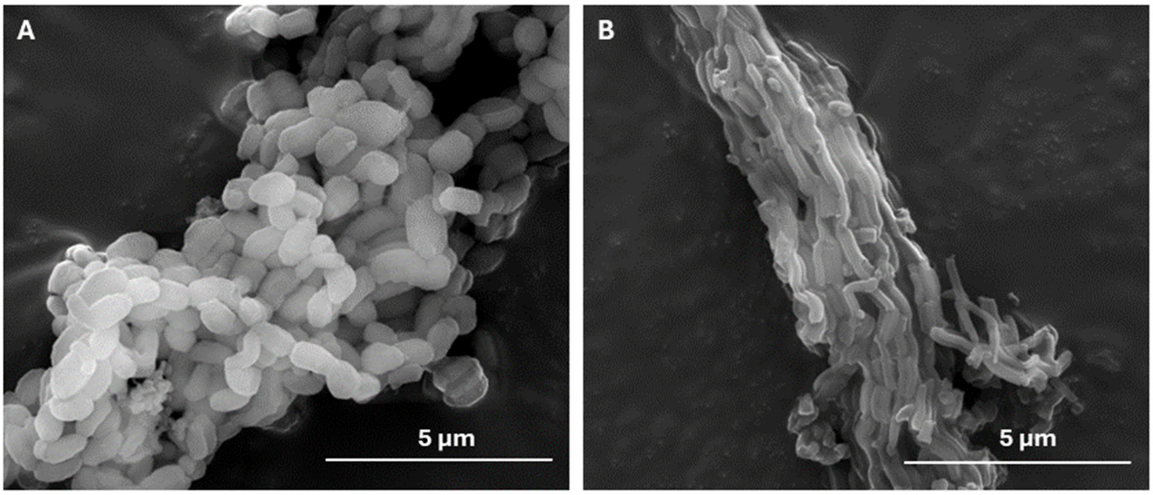


**Figure S3.** SEM images obtained for A) SBA-15 and B) *Aptes*SBA-15.

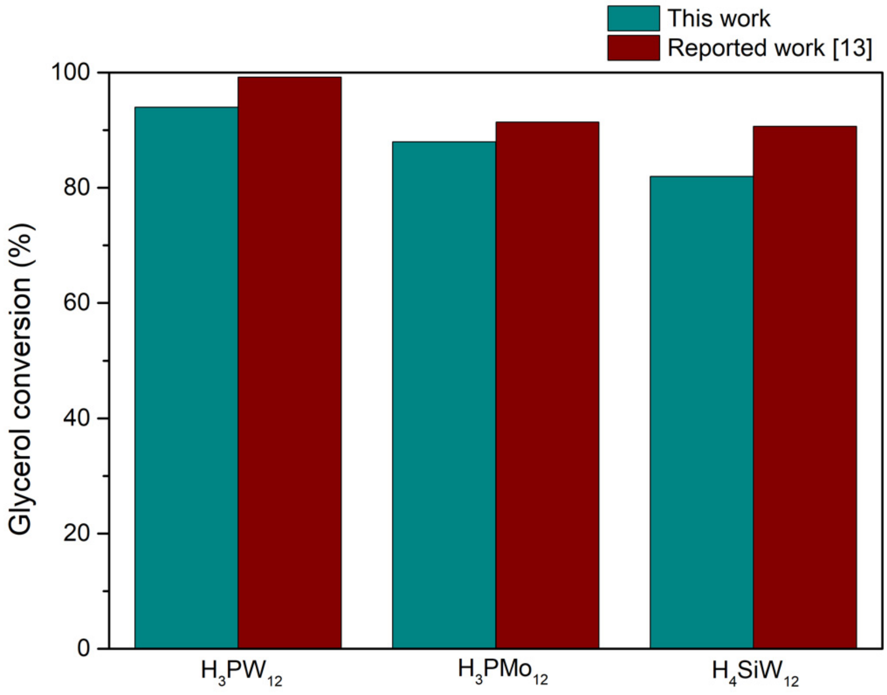


**Figure S4.** Comparison of the glycerol conversion results obtained using three different homogeneous catalysts ($H_3PMo_{12}$, $H_3PW_{12}$ and $H_4SiW_{12}$). The results of the reported work of Julião et al. (reference [13] in the manuscript) were obtained with 1:15 glycerol/acetone ratio, using 3 wt% of catalyst (compared to glycerol weight), at 25 °C after 5 min of reaction. The results of this work were obtained with 1:15 glycerol/acetone ratio, using a normalized catalyst acidity of 0.373 mmol $H^+$/g, at 25 °C after 5 min of reaction.

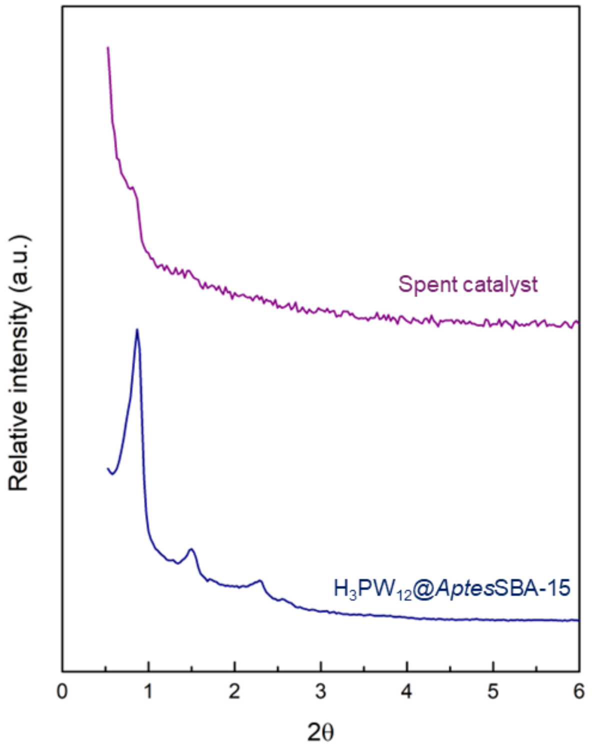


**Figure S5.** Powder XRD patterns for $H_3PW_{12}$@*Aptes*SBA-15 before catalysis and the spent $H_3PW_{12}$@*Aptes*SBA-15 catalyst after four catalytic cycles.

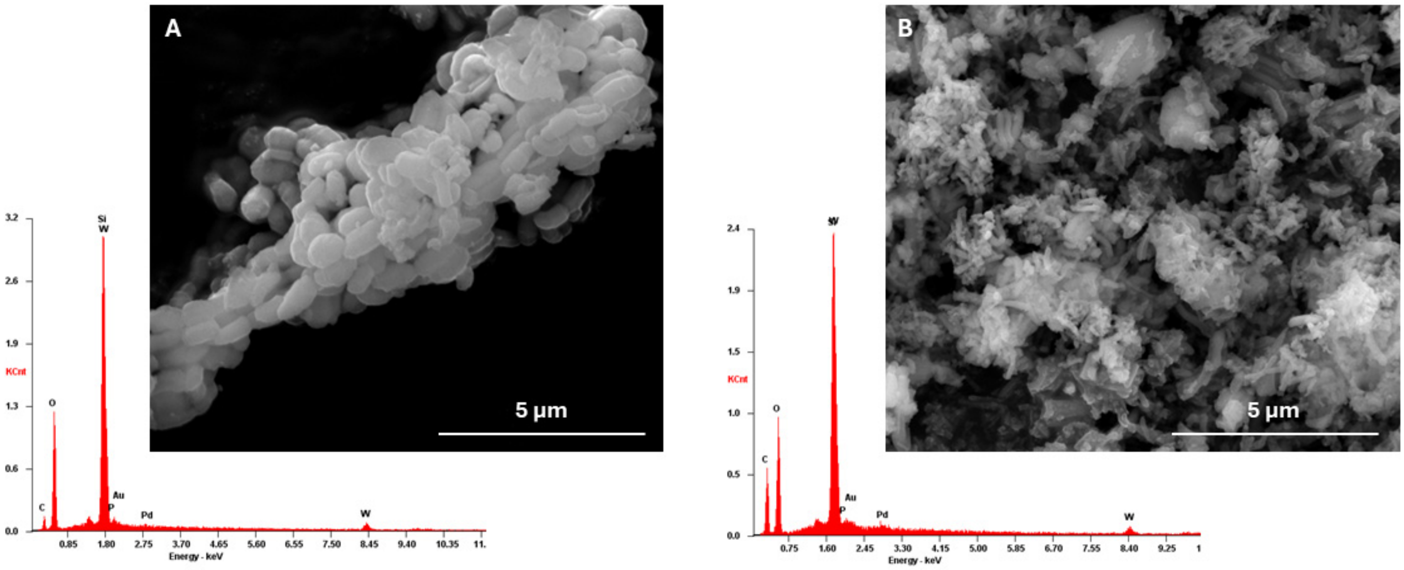


**Figure S6.** SEM images and EDS spectra obtained for A) $H_3PW_{12}$@*Aptes*SBA-15 before catalytic use and B) spent $H_3PW_{12}$@*Aptes*SBA-15, after four cycles.
